# Is Saccharin Injurious to Health?

**Published:** 1888-12-15

**Authors:** Const. Fahlberg

**Affiliations:** the Discoverer of Saccharin


### Is Saccharin Injurious to Health?

By Dr. Const. Fahlberg, the Discoverer of Saccharin.

In your issue of July 28th last you gave prominence to a statement to the effect that coal tar saccharin is injurious to the human system. The origin of this statement is doubtless traceable to the report issued by the Commission appointed by the Conseil d'Hygiene et de Salubrite de la France, to inquire into the properties of saccharin, and also to the remarks made by Dr. Worms at a meeting of the Académie de Médecine in Paris on April 10th last. I respectfully submit that the above report is objected to by all prominent men of science outside France who have been consulted in the matter, and who indeed express themselves in terms diametrically opposed to those contained in the report; and further, that the Government of France have not felt themselves impelled to take any action in consequence of the said report; that the words of Dr. Worms have become magnified and distorted by the French press until they no longer represent what he did say (*vide* his letter to the *Lancet* of the 10th instant); that this distortion is malicious, and is dictated by the fears of the French sugar industry lest sugar should be displaced (see the *Courrier de Lyons* of August 28th last, in which this charge is unhesitatingly made). That the words of Dr. Worms as uttered are opposed to the views of such authorities as Dr. Pavy, Dr. Stevenson, and others; that Dr. Worms does not occupy a high position in the scientific world as compared with those who differ from him upon this question; and that no medical man outside France has ever publicly declared himself against the use of saccharin. I have delayed replying to the charges which have been made against saccharin pending the completion of experiments impartially conducted with a view to test their veracity. Had they been confirmed, I should have discontinued the production or sale of saccharin. In view, however, of the fact that the highest authorities in the world now deny that saccharin exercises any harmful influence upon the system, I feel that I shall not appeal in vain to your sense of justice in asking you, after perusal of the enclosed pamphlet, to give as great a publicity to the contradiction as you have done to the assertion that saccharin acts injuriously upon the human system.

Salbke, Westerhüsen.

